# Melatonin and Artificial Light: Effects on Maternal and Fetal Health During Pregnancy

**DOI:** 10.1155/ogi/4879544

**Published:** 2026-09-12

**Authors:** Tamires de Abreu Cosendey, Gabriela Costa Oliveira, Rafael Cupertino Muzzi, Marli do Carmo Cupertino

**Affiliations:** ^1^ Dinâmica College of the Piranga Valley (FADIP), Ponte Nova, Minas Gerais, Brazil; ^2^ Mater Dei Health Network, Belo Horizonte, Minas Gerais, Brazil; ^3^ Federal University of Viçosa (UFV), Viçosa, Minas Gerais, Brazil, ufv.br; ^4^ Department of General Biology, Federal University of Viçosa (UFV), Viçosa, Minas Gerais, Brazil, ufv.br

**Keywords:** artificial light, circadian rhythm, maternal–fetal health, melatonin, pregnancy

## Abstract

**Introduction:**

The placenta produces melatonin during pregnancy, a hormone essential for circadian regulation and maternal–fetal metabolic, hormonal, and immunological modulation. Throughout pregnancy, melatonin production increases progressively, being synthesized by both the pineal gland and the placenta. Since exposure to artificial light at night (ALAN) reduces pineal melatonin secretion, it is hypothesized that light pollution may negatively impact women’s health, maternal health, and fetal development due to the inhibition of both pineal and placental synthesis.

**Material and Methods:**

This study aimed to analyze the role of melatonin during pregnancy and the effects of exposure to ALAN on maternal–fetal health. The following search terms (“*blue* light” OR “ALAN” OR “melatonin”) AND (“pregnancy”) were applied to *Title/Abstract*, with the following filters: (i) clinical trials, randomized trials, cohort studies, case–control studies, and case reports; (ii) publications between 2015 and 2025; (iii) studies involving human subjects and nonhuman animal models; (iv) female population; (v) languages: English, Portuguese, or Spanish. Information from the selected articles was extracted and organized into tables, separately for studies in humans and animals (mice and rabbits).

**Results:**

Exposure to ALAN reduces melatonin production and is associated with adverse maternal–fetal outcomes, including reduced fertility, sleep disturbances, preterm birth, metabolic alterations, gestational diabetes, hypothyroidism, altered gut microbiota, mood symptoms, excessive gestational weight gain, fetal growth changes, and congenital heart defects. Animal studies demonstrate causal effects of melatonin disruption, including impaired offspring development, inflammation, altered metabolic and hormonal rhythms, and pregnancy complications, whereas melatonin supplementation shows protective effects. These findings highlight the maternal circadian–melatonin axis as a potential target for preventive strategies. Although evidence is insufficient to recommend melatonin supplementation, antenatal counseling may include circadian hygiene measures, such as reducing evening light and screen exposure, maintaining regular sleep schedules, and optimizing nighttime darkness. Light‐based interventions, including biodynamic lighting and blue‐light‐blocking glasses, remain promising but require further clinical validation.

**Conclusion:**

It is concluded that melatonin plays a central role in regulating pregnancy and that exposure to ALAN poses a risk to maternal–fetal health. Mitigation strategies, such as lighting that mimics natural light, the use of blue light‐blocking glasses, and the combination of light and sleep, may represent relevant preventive measures for maternal–fetal health.

**Highlights**
• Artificial light at night (ALAN) exposure reduces melatonin signaling during pregnancy and is associated with adverse maternal–fetal outcomes.
• Maternal ALAN exposure is linked to sleep disturbances, metabolic alterations, preterm birth, and changes in fetal growth and neonatal health.
• Animal models demonstrate that maternal melatonin disruption causally affects offspring development, endocrine regulation, and metabolic homeostasis.
• Melatonin supplementation shows protective effects against developmental alterations induced by maternal chronodisruption in experimental studies.
• Preserving circadian health through light hygiene strategies may represent a promising preventive approach during pregnancy, although clinical evidence remains limited.

## 1. Introduction

Melatonin, a hormone identified by Lerner in 1958, is primarily produced by the pineal gland. However, subsequent research demonstrates that its synthesis also occurs in other tissues, such as the retina, intestine, and immune system cells [1, 2]. During pregnancy, the placenta begins to produce melatonin autonomously, contributing to increased maternal circulating levels. Because melatonin is a small amphiphilic (lipophilic and hydrophilic) indoleamine, it readily crosses biological membranes, including the placenta, without requiring active transport or undergoing significant metabolic modification. Consequently, maternal melatonin freely enters the fetal circulation, where it serves as the primary endocrine signal conveying maternal photoperiodic and circadian information to the developing fetus [2, 3].

During most of gestation, the fetal SCN and endogenous circadian oscillatory system are functionally immature, and robust fetal melatonin rhythms are absent until late gestation or after birth. Therefore, fetal circadian entrainment depends predominantly on maternal rhythmic melatonin exposure. This signal is biologically relevant because melatonin receptors MT1 (MTNR1A) and MT2 (MTNR1B) are widely expressed in the placenta, myometrium, and multiple fetal tissues, where melatonin regulates circadian synchronization, antioxidant defenses, immune modulation, vascular function, cellular differentiation, and tissue maturation. Within the framework of the Developmental Origins of Health and Disease (DOHaD), maternal circadian disruption‐including exposure to artificial light at night (ALAN) may therefore impair melatonin signaling during critical developmental windows, contributing to fetal programming events that increase susceptibility to disorders later in life [3–5].

Exposure to ALAN has emerged as a growing public health concern, primarily due to its potential to disrupt circadian rhythms. Advancing urbanization and modern lifestyles have increased this exposure, impairing the nocturnal secretion of melatonin. Evidence suggests that ALAN may affect the endocrine system and is associated with sleep disorders, metabolic and cardiovascular diseases, and, more recently, pregnancy complications [6, 7]. Blue light emitted by electronic devices, such as cell phones and computers, is particularly harmful, as it inhibits production of melatonin, delays the onset of sleep, and causes misalignment between internal biological cycles [8]. These effects are particularly concerning for vulnerable populations, such as pregnant women and children, who are more susceptible to circadian dysregulation. Rapid urbanization, coupled with behavioral changes resulting from the COVID‐19 pandemic—which has increased screen time due to the rise in remote learning and telework—has reduced the time spent in dark environments, amplifying the effects of ALAN [9]. Such changes may negatively impact the health of the mother and fetus, reinforcing the importance of investigating this phenomenon in a context of increasing urbanization [10, 11].

Despite growing recognition of the effects of ALAN on human health, studies specifically investigating its impacts during pregnancy remain limited. Thus, it is essential to understand how exposure to artificial light interferes with melatonin production and transfer during pregnancy. From a scientific perspective, the study contributes to the interdisciplinary field of environmental health, endocrinology, and maternal–fetal medicine by evidence and identifying gaps in knowledge. From a practical and social perspective, the results can inform public policies, guide clinical guidelines for pregnant women, and promote awareness campaigns regarding the risks of nocturnal exposure to artificial light. Considering the increasing prevalence of light pollution and electronic device use, understanding the relationship between melatonin disruption, ALAN exposure, and maternal–fetal health is clinically and socially relevant. This study aims to evaluate the role of melatonin during pregnancy and the potential effects of artificial light exposure on maternal and fetal outcomes by integrating evidence from human studies and experimental models.

## 2. Material and Methods

The search was performed in the *PubMed/MEDLINE* and *Google Scholar* databases, supplemented by an analysis of the references of the selected articles (*snowballing*). This approach allowed us to identify and synthesize the available evidence on melatonin and the effects of exposure to artificial light and their implications for maternal–fetal health, thereby contributing to answering the proposed research question.

### 2.1. Guiding Questions

The main questions to be answered in this review were as follows: (i) What is the role of melatonin in maternal health and fetal development during pregnancy? (ii) How does exposure to artificial light during pregnancy influence maternal and fetal melatonin levels? (iii) What changes in maternal–fetal health may occur due to reduced melatonin levels? (iv) Is there an association between exposure to artificial light and adverse maternal–fetal health outcomes? (v) What strategies can minimize the adverse effects of artificial light on maternal–fetal health?

### 2.2. Search Strategy

The literature search on the impacts of exposure to artificial light during pregnancy was conducted in the *PubMed/MEDLINE* and *Google Scholar* databases in two stages: (i) direct search for articles in the selected databases and (ii) indirect search in the reference lists of the included studies and websites, using the *snowballing* strategy.

The following search terms (*“blue* light” OR “ALAN” OR “melatonin”) AND (“pregnancy”) were applied to *Title/Abstract*, with the following filters: (i) clinical trials, randomized trials, cohort studies, case–control studies, and case reports; (ii) publications between 2015 and 2025; (iii) studies involving human subjects and nonhuman animal models; (iv) female population; (v) languages: English, Portuguese, or Spanish.

### 2.3. Selection Criteria

The following were defined as inclusion criteria: (i) original studies; (ii) studies that investigated exposure to artificial light during pregnancy in relation to melatonin levels and the repercussions for maternal health and fetal development, or that examined melatonin variation in the context of pregnancy; (iii) study designs such as clinical trials, randomized trials, cohort studies, case–control studies, and case reports; (iv) published between 2015 and 2025; (v) conducted with humans or animal models (mice and rabbits).

The population of interest comprises pregnant women and their fetuses, including studies on exposure to artificial light or melatonin variation related to maternal–fetal outcomes. The outcomes analyzed include melatonin levels, physiological changes, hormonal effects, circadian rhythm homeostasis, sleep quality, and maternal or perinatal complications, such as gestational disorders, preterm birth, and fetal size.

Studies that did not address exposure to artificial light during pregnancy or melatonin variation in the gestational context, investigations in animal populations other than mice or rabbits, studies irrelevant to the research question, review articles, conference abstracts, and letters to the editor were excluded. Additionally, information was extracted from studies that proposed strategies to mitigate potential adverse effects of artificial light exposure during pregnancy.

### 2.4. Data Extraction

Information from the selected articles was extracted and organized into tables, separately for studies in humans and animals (mice and rabbits). An independent reviewer (TAC) extracted the essential data, organized into descriptive levels, as detailed below:

For humans, the table columns included:

i. Source/country: lead author and year of publication; country where the study was conducted.
ii. n: number of study participants.
iii. Population: study group.
iv. Trimester: gestational period of the participants.
v. Study type: methodological design.
vi. Analyses: statistical or laboratory methods used.
vii. Sample characteristics: details of the sample, including age, health status, and inclusion/exclusion criteria.
viii. Duration: follow‐up or intervention period.
ix. Effect: observed impact of the ALAN intervention or exposure, described as positive (+) or negative (−).
x. Outcomes: main conclusions or final measures of the study.

For animals (mice and rabbits), the columns included:

i. Source: lead author and year of publication.
ii. n: number of animals included in the study.
iii. Sample: animal characteristics, such as species, sex, age, or weight.
iv. Study design: type of experiment conducted.
v. Analyses: laboratory or experimental methods used.
vi. Sample characteristics: additional details, such as number of offspring per mother, diet, or environmental conditions.
vii. Duration: duration of exposure or follow‐up.
viii. Clinical aspects: behavioral or physiological changes observed in the animals.
ix. Effect: observed impact of the ALAN intervention or exposure, described as positive (+) or negative (−).
x. Outcomes: final measures or conclusions of the study.

Any discrepancies in the extracted data were discussed and resolved jointly with two additional reviewers (MCC and GCO). No formal assessment of risk of bias was performed, given the methodological heterogeneity of the included studies.

## 3. Results

### 3.1. Study Selection

The search strategy yielded 115 studies (PubMed/Medline: 27; Google Scholar: 88). After removing 21 duplicates and 5 review articles, 73 studies were excluded for having inappropriate topics, as identified by reading the titles and abstracts. Sixteen articles were evaluated in *full text*, of which 4 were excluded for failing to meet the eligibility criteria. Additionally, 13 further articles were identified through indirect searches of the reference lists of the included studies and on websites, using the *snowballing* strategy. Thus, 25 studies were included.

### 3.2. Characteristics of the Included Studies in Humans and Animals

The information extracted from the 25 included studies was organized into tables, separately for humans and animals (mice and rabbits). The animal evidence covers pinealectomy, constant light, and dim ALAN, but not the chronic photoperiod shifting (CPS) model repeated shifts of the light:dark cycle that mimic shift work and jet lag in pregnant rodents. The pinealectomy, including constant light, and dim ALAN, and CPS, has emerged as one of the most translational experimental models of gestational chronodisruption because it reproduces the repeated phase advances and delays experienced by rotating shift workers and individuals with chronic jet lag. In this model, pregnant rodents undergo recurrent reversals of the light–dark cycle every 3–4 days throughout gestation. CPS induces persistent fetal programming effects involving metabolic dysfunction, adiposity, insulin resistance, adrenal clock dysregulation, altered steroidogenesis, renal dysfunction, hypertension, and behavioral abnormalities in adult offspring, thereby providing compelling evidence that maternal circadian misalignment alone can program long‐term offspring health [11, 12].

**FIGURE 1:**
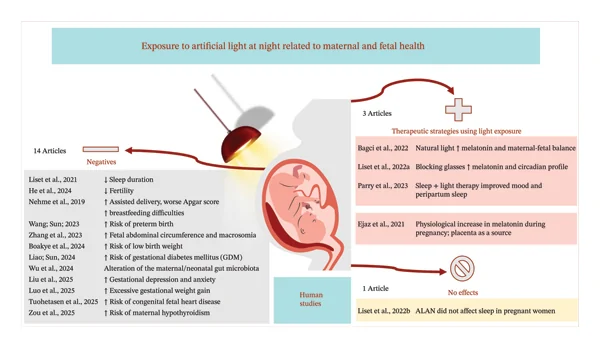
Effects of artificial light at night (ALAN) on maternal and fetal outcomes in human studies.

**FIGURE 2:**
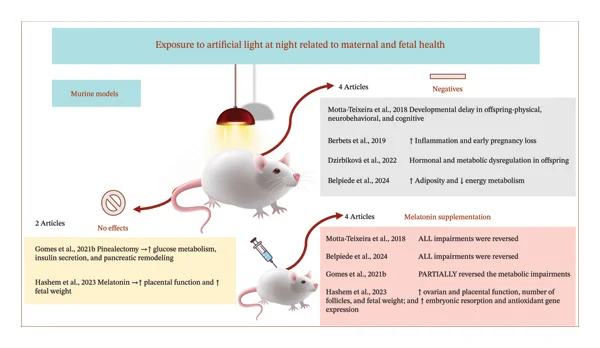
Effects of artificial light at night (ALAN) and melatonin on maternal and offspring outcomes in murine models.

For humans, the following were recorded: source (author and year), number of participants, population, gestational trimester, study type, analyses performed, sample characteristics, duration, effect of the ALAN intervention or exposure (+/−), and primary outcomes. For animals, the following were included: source, number of participants and population, study design, analyses performed, sample characteristics, duration, clinical aspects observed, effect (+/−), and final outcomes. Among the 25 studies, 19 were conducted in humans (Supporting table S1 and Figure 1) and 6 in animal models, including mice and rabbits (Supporting table S2 and Figure 2).

The included studies (2019–2025) evaluated the effects of ALAN exposure and melatonin disruption on reproductive, maternal, and neonatal health across different populations, mainly from China, Europe, Brazil, and the USA. Sample sizes ranged from 9 to 81,820 participants, including healthy pregnant women, women with pregnancy complications, night‐shift workers, and neonatal cohorts.

Of the 19 human studies included in this review, 7 (7/19) analyzed healthy pregnant women, 4 (4/19) investigated nulliparous pregnant women, 2 (2/19) evaluated women in the first trimester (2/19) of pregnancy using blue light‐blocking glasses, 2 (2/19) directly analyzed the risk of preterm birth, 2 (2/19) analyzed fetal size, 2 (2/19) included pregnant women and/or postpartum women with depression and/or anxiety, and 2 (2/19) focused on pregnant women with gestational diabetes. In addition, one study (1/19) investigated pregnant women working night shifts, 1 (1/19) evaluated pregnant women undergoing elective cesarean section, 1 (1/19) studied hospitalized pregnant women with healthy fetuses, 1 (1/19) evaluated gestational fertility, 1 (1/19) analyzed pregnant women and newborns regarding the diversity and composition of the gut microbiota, 1 (1/19) investigated pregnant women with gestational weight gain, 1 (1/19) analyzed pregnant women with fetuses with congenital heart defects, and 1 (1/19) included pregnant women with hypothyroidism.

The mean maternal age in the studies ranged approximately from 27 to 33 years, with a predominance of pregnant women without serious comorbidities, who were nonsmokers, abstained from alcohol, and had singleton pregnancies. In large‐scale Chinese studies, the mean age ranged from 27 to 32 years, with a majority of Han ethnicity, in addition to details on parity, education level, and specific obstetric conditions, lending greater methodological robustness to the samples.

Regarding study design, 6 (6/19) studies were prospective cohort studies, 4 (4/19) were retrospective cohort studies, 4 (4/19) were randomized clinical trials, 2 (2/19) observational case series/cross‐sectional studies, 2 (2/19) retrospective case–control studies, and 1 (1/19) cross‐sectional ecological study. Regarding the gestational period, 7 (7/19) studies included exclusively the third trimester, 4 (4/19) followed from the first to the third trimester, 2 (2/19) covered from the first to the third trimester and the postpartum period, 2 (2/19) evaluated from the pre‐pregnancy period through the postpartum period, and 1 (1/19) study covered the second to third trimesters, 1 (1/19) the first to second trimesters, 1 (1/19) the pre‐pregnancy period to the third trimester, and 1 (1/19) did not specify the period (aggregated population data by census tract).

Regarding the analyses performed, 12 (12/19) studies assessed exposure to ALAN, with eleven (12/19) using satellite imagery. 5 (5/19) studies analyzed melatonin and/or 6‐Ω levels, with 3 (3/19) using urinary parameters, 1 (1/19) using serum and placental assays, and 2 (2/19) using saliva samples. 5 (5/19) studies assessed sleep using actigraphy, scales, and/or sleep diaries. 2 (2/19) studies assessed mood/depression/anxiety using specific scales. One (1/19) analyzed fetal size via ultrasound and anthropometric outcomes at birth. One (1/19) study assessed low birth weight, with adjustments for green space coverage (NDVI). 1 (1/19) article investigated fertility using structured questionnaires and interviews at four time points (first, second, third trimester, and predelivery), with blood and urine collection. 2 (2/19) studies analyzed gestational diabetes, with diagnosis performed via an oral glucose tolerance test (OGTT). 1 (1/19) analyzed maternal and neonatal gut microbiota via 16S rRNA sequencing of stool samples. 1 (1/19) investigated gestational weight gain and excessive weight gain, using logistic and linear regression with analyses stratified by pre‐pregnancy BMI. 1 (1/19) assessed congenital heart defects confirmed by cardiac examinations and procedures, and 1 (1/19) study assessed hypothyroidism through laboratory tests of thyroid function. 3 (3/19) articles conducted interventions aimed at reducing the consequences of exposure to ALAN.

Regarding the effects of exposure to ALAN and the interventions tested, 14 studies (14/19) reported negative effects, 3 (3/19) observed positive effects, 1 (1/19) showed no significant effect, and 1 (1/19) study did not address the relationship with ALAN.

Regarding studies that reported positive effects or no effect, 4 (4/19) evaluated interventions to mitigate the effects of ALAN: 2 (2/19) used blue‐light‐blocking glasses, showing an improvement in the circadian melatonin profile or no significant effect on sleep, and 1 (1/19) applied a biodynamic lighting system, promoting an increase in nighttime melatonin. In addition, 1 (1/19) reported improved mood in pregnant women with peripartum depression through a combined sleep and light intervention.

Regarding negative effects on maternal–fetal outcomes, five studies (5/19) evaluated changes in melatonin: 3 (3/19) identified reduced melatonin secretion associated with ALAN, with a negative impact on maternal sleep, increased risk of preterm birth, and neonatal complications, while 2 (2/19) studies showed increased serum or placental melatonin in pregnant women, with a peak observed in the third trimester.

In addition to assessing metabolic, obstetric/fetal, psychological, and other biological outcomes associated with exposure to ALAN: 2 (2/19) on the risk of preterm birth, one (1/19) on increased fetal abdominal circumference and macrosomia, 1 (1/19) on low birth weight, 1 (1/19) on reduced fertility, 2 (2/19) to the increased risk of gestational diabetes mellitus, 1 (1/19) to changes in maternal gut microbiota, 1 (1/19) to an elevated risk of gestational depression and anxiety, 1 (1/19) to unhealthy weight gain during pregnancy, 1 (1/19) to an increased risk of fetal congenital heart disease, and 1 (1/19) to an increased risk of hypothyroidism, especially in pregnant women who were overweight prior to pregnancy.

According to Supporting Table S1, [13–39] small clinical studies demonstrated that pregnancy is associated with increased melatonin production, particularly during the third trimester, with the placenta acting as an important source of melatonin. Reduced nocturnal melatonin levels associated with nighttime light exposure or circadian disruption were linked to poorer sleep quality, increased risk of preterm birth, assisted delivery, lower Apgar scores, and breastfeeding difficulties [4, 13, 14].

Interventional studies suggest that preserving natural light‐dark cycles may improve maternal circadian regulation. Biodynamic lighting increased nocturnal melatonin production during hospitalization, while blue‐light‐blocking glasses improved melatonin profiles in pregnant women, although effects on sleep outcomes remain inconsistent [15–17]. Light‐based and sleep interventions also showed potential benefits for mood regulation in women with peripartum depression [18].

Large observational cohorts identified associations between ALAN exposure and multiple adverse outcomes. Higher ALAN exposure during pregnancy was associated with increased risk of preterm birth, altered fetal growth, macrosomia, low birth weight, gestational diabetes mellitus, gestational hypothyroidism, excessive gestational weight gain, maternal anxiety and depression symptoms, altered gut microbiota, and congenital heart defects [7, 19–27]. Additionally, pre‐pregnancy exposure to outdoor ALAN was associated with reduced fertility, suggesting possible effects on reproductive function before conception [28].

### 3.3. Characteristics of the Included Studies in Nonhuman Animals

We chose to include studies with nonhuman animals due to ethical and methodological limitations in studies with human pregnant women. Studies in nonhuman animals allow for more invasive interventions, such as pinealectomy, and detailed assessments of fetal development, especially regarding neurodevelopment, which would not be possible in humans.

Of the 6 (6/6) studies with nonhuman animals included, 4 (4/6) analyzed pregnant and/or lactating Wistar rats, 1 (1/6) evaluated nonpregnant and pregnant Wistar rats, and 1 (1/6) studied pregnant Hi‐Plus rabbits. Among the studies with Wistar rats, 3 (3/5) evaluated the offspring during the postnatal period, and all 5 (5/5) kept adult females on a 12:12 h light/dark cycle. In the study with Hi‐Plus rabbits, nulliparous adult females were monitored during pregnancy and their offspring during the postnatal period.

All 6 (6/6) studies were experimental. 3 (3/6) evaluated pregnant and/or lactating rats with pinealectomy and/or melatonin replacement; 1 (1/6) evaluated pregnant rats exposed to constant light (24 h/0 h) and nonpregnant rats for pineal gland analysis; 1 (1/6) evaluated pregnant rats exposed to dim artificial light; and 1 (1/6) evaluated pregnant Hi‐Plus rabbits administered melatonin during the first and second weeks of gestation.

Regarding the effects of exposure to ALAN and/or dim light, four studies (4/6) reported negative effects, and 2 (2/6) studies did not address the relationship with ALAN.

Three studies (3/6) evaluated pregnant and/or lactating rats that underwent pinealectomy and/or melatonin replacement; of these, 1 (1/3) demonstrated that melatonin deficiency delayed the physical growth, neurobehavioral development, and cognitive development of the offspring, 1 (1/3) impaired maternal metabolic adaptation and pancreatic *β*‐cell function, and 1 (1/3) altered energy metabolism, adiposity, and thermoregulation in the offspring effects that were partially reversed by melatonin replacement. One study (1/6) evaluated pregnant rats exposed to constant 24 h/0 h light, showing reduced maternal serum melatonin, morphological changes in the pineal gland, increased IL‐6, and early termination of pregnancy. Another study (1/6) investigated pregnant rats exposed to dim artificial light (< 2 lx) during pregnancy, showing disruption of the development of circadian rhythms of hormones and metabolites in the offspring, suggesting an endocrine‐disrupting effect. In the study with Hi‐Plus rabbits (1/6), melatonin administration during the first half of pregnancy increased follicle count, fetal and embryonic weight, placental efficiency, and the expression of antioxidant and regulatory genes, in addition to improving estradiol and progesterone levels, with effects dependent on the sensitive window of application.

According to Supporting Table S2, experimental animal studies provide mechanistic evidence that maternal melatonin is essential for pregnancy adaptation, placental function, and offspring development. In rodent models, maternal melatonin deficiency caused by pinealectomy or exposure to constant light resulted in impaired offspring growth, delayed neurobehavioral and cognitive development, altered glucose metabolism, impaired pancreatic remodeling, reduced insulin secretion, and disrupted energy homeostasis. These effects were largely prevented or reversed by melatonin replacement, demonstrating the protective role of maternal melatonin during gestation and lactation [29–31].

Exposure to ALAN in pregnant rats reduced melatonin secretion, induced pineal gland dysfunction, increased inflammatory responses (particularly IL‐6), and was associated with pregnancy loss [32]. Even low‐intensity nighttime light exposure altered the development of offspring circadian, hormonal, and metabolic rhythms, indicating that gestational ALAN may act as an endocrine disruptor affecting fetal programming [33].

Melatonin supplementation studies further demonstrated beneficial effects on reproductive and placental outcomes. In rabbits, melatonin administration during early pregnancy improved ovarian and placental function, increased fetal growth, reduced embryonic loss, and enhanced antioxidant gene expression, with the second week of gestation identified as a critical period for placental effects [34].

## 4. Discussion

The present review is, to our knowledge, the first to comprehensively evaluate the relationship between ALAN, melatonin disruption, and maternal–fetal health by integrating human and experimental evidence. The findings indicate that ALAN exposure may impair maternal melatonin signaling and contribute to adverse pregnancy outcomes through disruption of circadian regulation, placental physiology, and fetal development. Since melatonin represents a major endocrine signal connecting the maternal circadian system with the developing fetus, alterations in its secretion may compromise physiological synchronization during critical developmental windows [4, 6, 35, 36].

Beyond its chronobiotic function, melatonin regulates placental homeostasis, oxidative balance, inflammation, and fetal maturation. Through MT1 and MT2 receptor signaling, expressed in placental and fetal tissues, melatonin influences trophoblast differentiation, vascular function, mitochondrial activity, and endocrine regulation [36, 37]. Its antioxidant and anti‐inflammatory effects, including reactive oxygen species scavenging, activation of Nrf2‐dependent defenses, and suppression of NF‐*κ*B signaling, are particularly relevant to pregnancy complications characterized by oxidative stress and inflammation, such as preeclampsia, placental insufficiency, intrauterine growth restriction, and preterm birth [6, 36].

ALAN‐induced melatonin suppression may also affect peripheral circadian regulation through alterations in clock‐gene pathways, including BMAL1, CLOCK, PER1/2, and CRY1/2, which regulate metabolism, mitochondrial function, endocrine signaling, and cellular differentiation [6, 35]. These molecular changes provide a mechanistic link between maternal chronodisruption and fetal programming. Within the DOHaD framework, transient circadian disturbances during pregnancy may induce persistent modifications in offspring physiology, increasing susceptibility to metabolic, cardiovascular, endocrine, and neurodevelopmental disorders later in life [6, 35].

Human studies associate ALAN exposure with multiple adverse outcomes, including impaired sleep, preterm birth, altered fetal growth, gestational diabetes mellitus, hypothyroidism, changes in maternal gut microbiota, psychological symptoms, excessive gestational weight gain, low birth weight, and congenital heart defects [7, 14, 19–28, 38]. Although these studies are mainly observational, their findings are biologically plausible considering melatonin’s role in regulating metabolism, inflammation, oxidative stress, and placental function. In summary, human evidence indicates that ALAN exposure may affect maternal–fetal health through disruption of melatonin secretion, circadian regulation, metabolic pathways, and endocrine function. However, most available studies are observational, and further research is needed to establish causality, clarify dose–response relationships, and determine effective preventive interventions during pregnancy.

Experimental models provide additional evidence supporting causality. Maternal melatonin deficiency delays offspring physical growth, neurodevelopment, and cognition, effects that can be attenuated by melatonin replacement [29]. Constant light exposure during pregnancy induces pineal dysfunction, inflammatory activation, altered glucose regulation, and pregnancy complications [30, 32, 33]. In addition, CPS, which mimics repeated circadian disruption experienced by shift workers, demonstrates that maternal circadian misalignment alone can induce persistent alterations in offspring metabolism, adrenal function, and peripheral clocks [39, 40]. These findings strengthen the concept that maternal circadian disruption acts as an environmental programming factor during development. In summary, animal models support a causal relationship between maternal circadian disruption, reduced melatonin signaling, and adverse developmental outcomes. These studies indicate that melatonin regulates placental physiology, metabolic adaptation, oxidative balance, inflammatory responses, and long‐term offspring health, providing biological support for the associations observed in human studies. However, translation of these findings to clinical practice requires further investigation.

Epigenetic regulation may contribute to the persistence of these effects. Melatonin influences DNA methylation, histone modifications, and microRNA pathways involved in circadian regulation, oxidative stress, inflammation, and metabolism [6, 35]. Such mechanisms provide a plausible explanation for how temporary gestational exposures may produce long‐lasting changes in offspring phenotype within the DOHaD framework.

From a clinical perspective, these findings highlight circadian health as a potentially modifiable factor in prenatal care. Current evidence does not support routine melatonin supplementation during pregnancy; however, antenatal counseling may include strategies to preserve endogenous melatonin production, such as reducing unnecessary nighttime light exposure, limiting evening screen use, maintaining regular sleep schedules, and optimizing bedroom darkness. For pregnant women exposed to occupational circadian disruption, including night‐shift workers, interventions aimed at reducing circadian misalignment and protecting daytime sleep may be particularly relevant. Biodynamic lighting has shown potential to preserve nocturnal melatonin production, whereas blue‐light‐blocking glasses remain promising but require further validation before routine clinical recommendation [15].

Experimental studies suggest that melatonin supplementation may protect against developmental alterations induced by maternal chronodisruption, improving placental function, metabolic adaptation, and offspring development [29, 31, 34]. However, clinical trials are still required to establish the safety and efficacy of supplementation during pregnancy. Overall, current evidence supports a model in which ALAN suppresses maternal melatonin signaling, disrupts maternal–fetal circadian communication, and contributes to fetal programming through endocrine, metabolic, inflammatory, and epigenetic mechanisms. Future research should determine whether targeted light‐based interventions can improve maternal and neonatal outcomes.

## 5. Conclusion

The placenta is an important source and target of melatonin during pregnancy, and maternal melatonin regulates circadian, metabolic, immune, and developmental processes essential for maternal–fetal homeostasis. Exposure to ALAN suppresses melatonin signaling and is associated with adverse maternal, fetal, and neonatal outcomes, including sleep disturbances, metabolic alterations, obstetric complications, and offspring developmental changes. Experimental studies support causal relationships between maternal melatonin disruption and fetal programming, while melatonin supplementation shows protective effects.

Current evidence supports a model in which ALAN disrupts maternal–fetal circadian communication, impairs placental antioxidant and anti‐inflammatory pathways, alters clock‐gene regulation, and contributes to developmental programming within the DOHaD framework. However, limitations in human studies, including exposure assessment, melatonin measurement, and confounding factors, highlight the need for further research. Future multidisciplinary studies should clarify molecular mechanisms and evaluate preventive strategies.

Although interventions such as biodynamic lighting and blue‐light‐blocking glasses show potential, their clinical application during pregnancy requires further validation. Reducing unnecessary nighttime light exposure and promoting healthy sleep environments may represent practical strategies to support maternal–fetal health and inform future clinical recommendations and public health policies.

## Funding

No external funding was received; the study was supported by internal resources.

## Conflicts of Interest

The authors declare no conflicts of interest.

## Supporting Information

Additional supporting information can be found online in the Supporting Information section.

## Supporting information


**Supporting Information** Supporting Table S1—Methodological and clinical characteristics of the human studies included in the review, presenting author/year and country, number of participants, population, gestational trimester, analyses performed, sample characteristics, study duration, and main outcomes. Supporting Table S2—Methodological and experimental characteristics of the studies in nonhuman animal models included in the review, presenting author/year, number and type of animals, experimental design, analyses performed, sample characteristics, study duration, clinical aspects observed, effects of ALAN, and main outcomes.

## Data Availability

Data sharing is not applicable to this article as no new data were created or analyzed in this study.
