# Supplementary material for: Melatonin and Artificial Light: Effects on Maternal and Fetal Health During Pregnancy
Source: Obstet Gynecol Int. 2026 Sep 12;2026:4879544. doi: 10.1155/ogi/4879544 (PMC13570495; doi:10.1155/ogi/4879544)
Supplement: Supplementary file 1 [file OGI-2026-4879544-s001.docx]

**Supplementary Table S1** – Methodological and clinical characteristics of the human studies included in the review, presenting author/year and country, number of participants, population, gestational trimester, analyses performed, sample characteristics, study duration, and main outcomes.

| **Source/**  **Country** | **n** | **Population** | **Quarter** | **Analyses** | **Sample characteristics** | **Duration** | **Outcomes** |
| --- | --- | --- | --- | --- | --- | --- | --- |
| Nehme et al., 2019¹⁵  Brazil | 9 | GS, nurses (day shift n=6; night shift n=3) | 3rd | Melatonin (urine), maternal indicators, and neonatal outcomes | Age 30–37 years, no comorbidities | Follow-up for 10 days, and up to 2 months postpartum | Exposure to ALAN and nighttime habits during pregnancy reduce melatonin levels, which is associated with a higher risk of assisted delivery (cesarean section and forceps), lower Apgar scores, and difficulties with breastfeeding. |
| Ejaz et al., 2021⁴  United Kingdom | 71 | GNS (MEL-P, n=26), with elective cesarean section (MEL-P2, n=15), and non-pregnant controls (n=30) | 1st–3rd (MEL-P); 3rd (MEL-P2) | Melatonin (blood) and placental levels and maternal/obstetric outcomes. | Age 16–45 years, healthy fetuses | During pregnancy and 3–6 days before cesarean section and 24 h postpartum, and placenta 15 min postpartum | Melatonin levels rise during pregnancy, peaking in the third trimester, and drop sharply after delivery. These findings suggest that the placenta is an important source of melatonin with a physiological role in pregnancy. |
| Liset et al., 2021²⁰  Norway | 130 | GNS (n=61) and non-pregnant controls (n=69) | 3rd | Sleep and exposure to ALAN (actigraphy, sleep diary, and specific scales) | Pregnant women: mean age 30.6 years, no comorbidities | 1 week of continuous monitoring | Exposure to nighttime light was associated with shorter sleep duration. |
| Bagci et al., 2022¹⁸  Germany | 47 | Pregnant women hospitalized with healthy fetuses: room with BDL (n=27) and control (n=20) | 3rd | Light dosimetry, melatonin (urine), and daily questionnaires | Median age 30 years, with various obstetric complications | Assessment at 3 times: day 1, day 3, and day 5 of hospitalization | The BDL system with natural light at dawn increased nocturnal melatonin, suggesting that maintaining natural light promotes maternal-fetal melatonin homeostasis. |
| Liset et al., 2021³⁵  Norway | 60 | GNS wearing blue light-blocking glasses (BB, n=30) and wearing control glasses (n=30) | 3rd | Melatonin (saliva) and sleep (sleep diary) | Mean age 30.5 years. Night work, eye condition affecting translucency or, melatonin levels outside the normal range | 3 weeks (1-week baseline + 2 weeks of intervention)  . | Wearing blue-light-blocking glasses (orange lenses) at night increased melatonin and improved the circadian profile in pregnant women, compared to control glasses (gray, partial blocking). |
| Liset et al., 2022³⁸  Norway | 60 | GNS with blue light-blocking glasses (BB, n=30) and with control glasses (n=30) | 3rd | Sleep (actigraphy, sleep diary, and sleepiness scales) | Mean age 30.5 years, with somatic/psychiatric disorders, fever, night work, eye conditions affecting translucency, or melatonin levels outside the normal range | Blue light-blocking glasses (orange lenses) vs. control glasses (gray, partial blocking) for 3 weeks | Sleep assessment (objective and subjective) showing that ALAN did not affect sleep duration, efficiency, insomnia, or sleepiness in pregnant women. |
| Parry et al., 2023³⁶    USA | 89 | Pregnant women (n=21 healthy controls; n=16 with postpartum depression) and postpartum women (n=27 controls; n=25 with postpartum depression) | 3rd trimester and postpartum | Weekly structured interviews, mood scales, sleep (actigraphy), melatonin (urine) | Age 20–40 years, non-smokers, no bipolar disorder or use of drugs/medications that interfere with the circadian rhythm | 6-week bright white light intervention: in the evening, 90 min before bedtime, or in the morning, 30 min after waking | Sleep and bright white light interventions improved mood in pregnant women with peripartum depression (light at night) and in postpartum women with postpartum depression (light in the morning), associated with an advance in the melatonin phase, suggesting that chronotherapy interventions combining sleep and light are effective and safe. |
| Sun et al., 2023⁷  China | 2,850 | Control group (n = 2,375) and Preterm Birth (PTB) group (n = 475) | 1st–3rd | Exposure to ALAN (satellite) and preterm birth (<37 weeks) Obstetric records/medical charts | Mean age 31–32 years, singleton pregnancy | Exposure calculated throughout the entire pregnancy | ALAN exposure during the first and second trimesters was associated with the risk of preterm birth. |
| Wang et al., 2023²¹  China | 50 | Pregnant women at risk of preterm birth (TPL, n=20) and normal pregnant women (NPW, n=30) | 3rd | Sleep (actigraphy and diary), melatonin and cortisol (saliva), and maternal indicators (mood questionnaires) | Mean age 33 years, predominantly nulliparous women. Diagnosis of TPL at hospital admission | Data collection over 2 days | Lower melatonin secretion associated with risk of preterm birth and poorer sleep quality in the third trimester. |
| Zhang et al., 2023¹⁹  China | 6,210 | Pregnant women | 2nd–3rd trimester through delivery | Exposure to ALAN (satellite) and neonatal outcomes (fetal ultrasound, and birth anthropometry) | Mean age 30 years, mostly nulliparous | Period from 2015 to 2021 | Higher exposure to ALAN, especially in the second trimester, was associated with increased fetal abdominal circumference and risk of macrosomia, with a more pronounced effect in female fetuses. |
| Boakye et al., 2024²⁹  USA | 7,808 | Single live births (2009–2015) | Unspecified | Exposure to ALAN (satellite) and low birth weight (obstetric record) | Secondary data from official health records | 2009–2015 | Association between ALAN and low birth weight. |
| He et al., 2024²²  China | 3243 | Pregnant women who conceived naturally, with residential address data available | Prenatal and postnatal periods and 1st–3rd | Exposure to ALAN (satellite), melatonin (blood and urine), and reproductive indicators (subfertility) | Mean age 29 years. Spontaneous pregnancy, <28 weeks at study enrollment | Year prior to pregnancy; monthly weighted average by residential address | Pre-pregnancy exposure to outdoor ALAN was associated with reduced fertility, highlighting the potential effects of light pollution on women’s reproductive health. |
| Liao et al., 2024²³  China | 9,120 | Pregnant women with gestational diabetes (GDM, n = 1,484) and without gestational diabetes (n = 7,636) | 1st–3rd trimester and postpartum | Exposure to ALAN (satellite) and diagnosis of gestational diabetes (OGTT) | Mean age 27–29 years, pre-pregnancy BMI 20.9–22.04 | During pregnancy (1st and 2nd trimesters: analysis of exposure to ALAN); in the 3rd trimester and 24 h postpartum | High levels of ALAN exposure during pregnancy may be associated with an increased risk of gestational diabetes mellitus. |
| Sun et al., 2024²⁴  China | 5,720 | Pregnant women with gestational diabetes mellitus (GDM) (n=1,430) and controls (n=4,290) | 1st–2nd | Exposure to residential ALAN (satellite) and diagnosis of gestational diabetes (OGTT) | Age 30–32 years, nulliparous and multiparous women; mean pre-pregnancy BMI 20.7–21.3 kg/m²; singleton pregnancies | Exposure during 1st and 2nd trimesters, GDM outcome between 24–28 weeks | Exposure to outdoor ALAN during pregnancy is associated with an increased risk of GDM. |
| Wu et al., 2024²⁶  China | 72 | Pregnant women (n = 44) and newborns (n = 28) | 3rd | Exposure to ALAN (satellite), maternal and neonatal microbiota (feces), and maternal/neonatal indicators (questionnaires) | Mean maternal age 30 years, ≥28 weeks’ gestation | Data from 2017 to 2019; follow-up until delivery (neonatal sample collection within 48 hours) | Positive association between exposure to ALAN during pregnancy and the composition of the maternal gut microbiota. |
| Liu et al., 2025²⁷  China | 1047 | Pregnant women/postpartum women with depression (62); without depression (985); with anxiety (128); without anxiety (919) | Prenatal and postnatal and 1st–3rd | Exposure to ALAN (satellite) and symptoms of depression and anxiety (scales) | Gestational age 24 and 28 weeks of gestation; age ≥18 years, mean 30 years | Recruitment between 2021 and 2022; post l follow-up up to 2 years postpartum in the general cohort study | Exposure to high levels of artificial outdoor light (ALAN) three months before pregnancy and during the first trimester is a risk factor for symptoms of depression and gestational anxiety. |
| Luo et al., 2025²⁸  China | 51,096 | Pregnant women with normal (30,600) and excessive (20,496) gestational weight gain | Pre-pregnancy and 1st–3rd | Exposure to ALAN (satellite) and normal/excessive gestational weight gain (medical records) | Ages 18–50 years, mean 29–30 years, singleton pregnancy | Followed from conception through the end of pregnancy (2013–2022) | Exposure to ALAN before and during pregnancy was associated with an increased risk of weight gain. These effects were stronger in women with normal pre-pregnancy weight. |
| Tuohetasen et al., 2025³⁰  China | 18,252 | Pregnant women with fetuses with congenital heart defects (n = 9,764) and healthy controls (n = 8,488) | 1st–3rd | Exposure to ALAN (satellite) and congenital heart defects (diagnosed by tests) | Maternal age > 18 years. ≥35 years (15% in cases vs. 10% in controls) | Full gestational period (births between 2004–2022) | Exposure to ALAN during pregnancy is associated with an increased risk of congenital heart disease (CHD) in fetuses. This association was observed in nearly all subtypes of CHD, particularly in severe cases. |
| Zou et al., 2025²⁵  China | 81,820 | Pregnant women with hypothyroidism (n = 3,902), without hypothyroidism (n = 77,918) | 1st–3rd | Exposure to ALAN (satellite) and thyroid function (laboratory tests) and maternal indicators (questionnaires) | Mean age 29–30 years, pre-pregnancy BMI 21.7–22 kg/m² | From recruitment (6–13 weeks) through postpartum | High exposure to external ALAN is associated with an increased risk of hypothyroidism in pregnant women, especially in those who were overweight before pregnancy. |

**Legend:** ALAN: *artificial light at night*; BB: *blue-blocking* glasses; BDL: *biodynamic lighting*; CHD: *congenital heart* disease; GDM: gestational diabetes mellitus; GNS: healthy nulliparous pregnant women; GS: healthy pregnant women; BMI: body mass index; MEL-P: pregnant women during pregnancy; MEL-P2: pregnant women undergoing elective cesarean section; NPW: *normal pregnant women*; OGTT: *oral* glucose tolerance test; PTB: *preterm birth*; TPL: *threatened preterm labor*.

**Supplementary Table S2** – Methodological and experimental characteristics of the studies in non-human animal models included in the review, presenting author/year, number and type of animals, experimental design, analyses performed, sample characteristics, study duration, clinical aspects observed, effects of ALAN, and main outcomes.

**NON-HUMAN ANIMAL MODELS**

| **Source** | **n** | **Sample** | **Design** | **Analyses** | **Sample characteristics** | **Duration** | **Clinical presentation** | **Outcomes** |
| --- | --- | --- | --- | --- | --- | --- | --- | --- |
| Motta-Teixeira et al., 2018³¹ | 78  rats | Lactating Wistar rats and their offspring | CTL (control, normal melatonin); PINX (pinealectomized absence of melatonin); PINX + MEL (pinealectomized with melatonin replacement) | Somatic and physical growth (such as weight gain, fur, eyes, teeth, genitalia), neurobehavioral development, cognition, and hippocampal neurogenesis | Pregnant females (8 weeks), with or without pineal melatonin rhythm, maintained on a 12:12 h light/dark cycle; pups assessed postnatally | Pregnancy and lactation | Impact of melatonin deficiency during gestation and lactation on the physical growth, neurobehavioral development, and cognitive function of offspring, and the effects of therapeutic replacement | Maternal melatonin deprivation delayed the onset of physical characteristics, neurodevelopment, and cognition in male offspring, while supplementation prevented these deficits, suggesting implications for pregnant women exposed to ALAN. |
| Berbets et al., 2019³² | 52  rats | Pregnant and non-pregnant Wistar rats | Control: 12h/12h light/dark cycle (n=10); Exposed: 24h/0h constant light (n=14); additional groups of non-pregnant female rats (n=28) for morphological analysis of the pineal gland | Serum levels of melatonin and cytokines (IL-6, IL-10, TNF-α, IL-1β, IL-4); morphological and ultrastructural analysis of the pineal gland; assessment of gestational development | Pregnant adult females (17–24 weeks; 200–280 g), kept under white halogen lamps (6500 K, 1200 lm) 50 cm from the cages | Light exposure from the 3rd day after mating until the 24th day of gestation (21 days) | Effect of constant nighttime light on maternal melatonin, pineal gland function, pro-inflammatory response, and pregnancy progression | Continuous light exposure resulted in pineal dysfunction, reduced serum melatonin, an increased pro-inflammatory response (elevated IL-6), and early pregnancy termination. No exposed female carried the pregnancy to term. This highlights the association between pineal gland dysfunction, inflammatory response, and pregnancy progression under the influence of light, suggesting relevance to the growing incidence of human infertility related to overexposure to nighttime light. |
| Gomes et al., 2021³³ | 3–12 female rats per group | Pregnant and lactating Wistar rats | CTL (control, intact female rats), PINX (pinealectomized—melatonin-deficient), PMF (pinealectomized + fixed melatonin replacement), PMD (pinealectomized + dynamic melatonin replacement) | Glucose tolerance, insulin resistance, β-cell apoptosis, melatonin receptor (MT2) expression, pancreatic remodeling, reactive oxygen species (ROS), and antioxidant activity | Nulliparous females (8 weeks old), maintained on a 12:12 h light/dark cycle, temperature-controlled (23 ± 2°C), with free access to water and food, evaluated during pregnancy and lactation | Pinealectomy prior to pregnancy; melatonin replacement throughout pregnancy and lactation | Evaluation of the role of maternal melatonin in metabolic adaptation to pregnancy, beta-cell function, and pancreatic remodeling, and the effect of therapeutic melatonin replacement | The absence of maternal melatonin impaired glucose metabolism, insulin secretion, and pancreatic remodeling, while supplementation—especially with a dynamic profile—partially reversed these effects. The study demonstrates the regulatory role of melatonin in maternal metabolic adaptation during pregnancy. |
| Dzirbíková et al., 2022³⁴ | 28  rats | Pregnant Wistar rats and their offspring | CTRL - control (12:12 h light/dark cycle)  ALAN (exposure to dim artificial light <2 lx throughout gestation) | Circadian rhythms of melatonin, corticosterone, vasopressin, thyroid hormones (T3, T4), and metabolites (glucose, cholesterol, triacylglycerols) in pups (P3, P10, P20) | Pregnant adult females (8–9 weeks) maintained on a standard light/dark cycle or exposed to artificial nighttime light (<2 lx) during pregnancy; pups assessed up to the 20th postnatal day | Pregnancy and early postnatal period | Impact of exposure to artificial night light during pregnancy on the development of hormonal and metabolic rhythms in pups, suggesting an endocrine-disrupting effect | Exposure to ALAN during pregnancy disrupted the development of daily rhythms in measured hormones and metabolites, suggesting that ALAN during pregnancy may act as an endocrine disruptor, interfering with the normal development of offspring. |
| Hashem et al., 2023³⁷ | 80  rabbits  n = 20 per group): | Hi-Plus rabbits and their offspring | FW (melatonin 0.7 mg/kg/day, 1st week of gestation);  SW (melatonin 0.7 mg/kg/day, 2nd week of gestation);  F+SW (melatonin 0.7 mg/kg/day, 1st and 2nd weeks);  C (control, no melatonin) | Ovarian follicles, fetal resorption, fetal and embryonic sac weights, placental efficiency, hormonal profile (estradiol, progesterone), and expression of antioxidant and cell cycle regulatory genes | Clinically healthy, nulliparous adult females, maintained under standard husbandry and hygiene conditions | First half of gestation, evaluation on day 18—after euthanasia | Evaluation of the effects of melatonin administered during different sensitive periods of the first half of gestation on ovarian and placental function, hormonal profile, and reproductive outcomes | Melatonin improved ovarian and placental function, increased follicle count and fetal weight, reduced embryonic resorption, and optimized the expression of antioxidant genes. The second week of gestation was identified as a sensitive window for placental effects, while the first week primarily benefited ovarian function. |
| Belpiede et al., 2024³⁸ | 5–20 rats per group | Pregnant Wistar rats and their offspring | CTL (control pregnant rats);  PINX (pinealectomized pregnant rats);  PINX + MEL (pinealectomized pregnant rats with melatonin replacement)  Pups divided into C (control mothers), P (PINX mothers), and PM (PINX + MEL mothers) | Morphometric growth of offspring, energy metabolism, thermoregulation, food intake, adipose tissue deposition, insulin sensitivity, Ucp1 expression, mitochondrial complex activity | Nulliparous adult females (8 weeks), maintained on a 12:12 h light/dark cycle; male pups evaluated from birth until 12 weeks post-weaning; part of the offspring on a normal diet and part on a high-calorie diet | Pinealectomy prior to pregnancy; melatonin replacement during pregnancy and lactation; follow-up of offspring until 12 weeks post-weaning | Impact of the absence of maternal melatonin on energy metabolism, adiposity, blood glucose, thermoregulation, and response to nutritional challenge in the offspring; evaluation of the efficacy of melatonin replacement | Maternal melatonin deficiency impaired the offspring’s energy metabolism. Melatonin replacement normalized metabolism, allowing for functional adaptations (reduced food intake, improved thermoregulation), resulting in lower body weight gain and reduced white adipose tissue mass. |

# *Leg*end: ALAN: Artificial light *at night*; C: *Control*; CTRL: *Control*; FW: Melatonin administration during the *first week* of gestation; F+SW: Melatonin administration during *the first* and second weeks of pregnancy; MEL: Mel*atonin*; PINX: Pinealectomized; PMD: Pinealectomized + dynamic melatonin *replacement*; PMF: Pinealectomized + *fixed* melatonin replacement; SW: Melatonin administration in the *second week* of pregnancy.
